# Modern Sociology

**Published:** 1901-09-28

**Authors:** 


### Modern Sociology.

### THE CASE OF THE SHOP ASSISTANT.

By a Correspondent.

### IV.—The Need for Inspection.

It is an extraordinary thing that places in which people are brought together for purposes of retail trade should be so completely left to chance in the matter of sanitation ventilation, etc., as is the case with shops. We have regulations for the child in the elementary school, specifying the required number of cubic feet of air; the factory and workshop hand is similarly protected; and the occupants of lodging-houses and shelters are prevented from overcrowding at night. It seems sufficiently reasonable that all places where a number of people work and sleep should be thoroughly inspected, and we should like to see the principle in operation wherever human beings congregate. Certainly it is time that the habitation of the shop-assistants, numbering as they do upwards of a quarter of a million, should be brought under systematic inspection. "The manager who showed me over the rooms," says Miss Clara Collet, "had himself been a shop assistant in several shops. He considered that, from what he knew of the facts, sanitary inspection of shops and of living accommodation was most desirable." Miss Collet says "the difficulties of such inspection would, however, be very great, owing to the impossibility of fixing any standard of necessary comfort or cleanliness." But the difficulty has been met in the cases referred to above.

That the case demands investigation is undeniable. The health of the shop assistant suffers not only from the effects of long hours and bad meals, but also from the lack of fresh air. Sir William Church has spoken of the effects on health of confinement in a bad and contaminated air, and he attaches no importance to the statement that the work performed in such air is not necessarily arduous; what he insisted on before the Lords' Committee was the need of fresh air and sunlight. Long hours and bad atmosphere were inseparable, in his opinion. And it must be remembered that even if a shop is well ventilated, it is often crowded to such an extent that the air becomes contaminated in a very short time. Any lady who has taken part in a bazaar, say for three days, in a public building, knows what the atmosphere is like; and few would care to look forward to breathing it year in and year out. It is not surprising that, with an already lowered vitality, shop assistants should suffer from pulmonary disease, and that "incipient phthisis" should occur with painful frequency on the medical certificates of those who claim sick benefit. "Hardly a day passes," a shop-walker in a large West-End shop tells us, "without one of the young ladies fainting."

They are forbidden to leave the shop, and visit the sitting-rooms. In some cases these sitting-rooms may be all that could be wished; in one house we have seen sitting-rooms underground, where artificial light has always to be used, and where what little ventilation there is, is more a matter of chance than design. "We cannot sit there for more than five minutes without getting a headache," one assistant said. And apart from absence of light and air, these places are dismal in the extreme; a piano with broken keys resounds hollow to the touch, a few newspapers are on the table, and a portrait of the head of the firm beautifies the walls. There is a whole row of these sitting-rooms in which nobody sits.

In what is called a "good shop," the dining-room is underground and unventilated; the assistants hire a room with a piano elsewhere; and the fact seems to be that, at any rate in neighbourhoods where rents are high, the greater part of the house is devoted to gorgeous show-rooms, and the living accommodation is squeezed into the background. As to lavatories, "Washing," says an assistant of several years' experience, "is as superficial as the dress of many assistants; a surprising number of girls are of dirty habits to begin with, and the want of proper lavatory arrangements makes them very quickly deteriorate from even their low standard. The odour of unwashed feet was more than I could bear, and at last I struck and got lodgings outside." She added that, tired as the girls were with so many hours' work, it was not surprising that they had little energy left to make long journeys down to underground regions for jugs of water; and that if they used their allowance at night there would be none left for the morning. We asked if it was customary to provide baths; and an amused smile broke over her face.

There is a house where the assistants belonging to a firm in the West End live, where we saw beautiful and costly porcelain baths; and we have been told of another house where baths are provided in the proportion of one to every thirty assistants, "they are generally occupied when you arrive," our informant added. In one large firm the assistants go to the public baths.

Sleeping accommodation varies as greatly as other provisions for bodily comfort. In the good house before quoted many of the assistants have separate rooms, which are neatly and tastefully furnished, with plenty of cupboard room, and in which, when we visited them, the only unpleasing things were the beds themselves, which could only by a stretch of imagination be called "made." The girls were supposed to make them before going to business.

Not far from this house the young men are sleeping 24 in a room. Quiet and leisure are unknown, and one man tells us that he has only been able to read one book since he came to London some eighteen months ago. He says "life was worth living in the country."

Why do the assistants come to London? "West End experience" is the magnet that draws them; and many have regretted the step when it was too late. One very serious evil of this herding together is the contamination of boys fresh from a country home; and we have been told of more than one instance in which, in the course of a few months, a boy of sixteen has become more depraved than any of his associates.

There is a strong and growing feeling among the more thoughtful assistants that the living-in system is radically bad and should be abolished, and the following resolution was unanimously passed at demonstrations in Hyde Park and at Liverpool on Sunday afternoon, July 21st: "Believing the living-in system to be detrimental to the physical, mental, and moral well-being of those who by necessity have to accept it as a condition of employment—knowing also that it denies citizenship to thousands of adult men, and, therefore, politically and socially degrades them—seeing, too, that it is only retained because it enables the employers to make huge profits out of the 'board and lodging,' as well as the labour of those employed, and feeling the time has arrived when those engaged in distribution should be paid their wages in current coin of the realm, like other workpeople—this meeting calls upon all those affected to work for its total abolition."

"If shopkeepers knew that they could make more money by letting the upper part of their houses out as flats," says a shopkeeper, "they would abolish the living-in system to-morrow." A girl who is gaining her "West End experience" at greater expense than she anticipated, says, on this point: "It would be very well for 'fellows,' but many girls I know are too giddy to be left to provide for themselves in lodgings." This is the other side of the question, and one that the assistants themselves will have to answer, but in the meantime it may be pointed out that life in lodgings is not the only alternative. A scheme has been suggested, which seems quite within the limits of possibility, for erecting houses something on the model of the Rowton lodging-houses, with a public restaurant attached. In such a scheme all depends upon good management, but with this, and a Rowton to finance it at starting, there seems to be no reason why it should not be a success.

